# Response surface methodology for optimization of methylene blue adsorption onto carboxymethyl cellulose-based hydrogel beads: adsorption kinetics, isotherm, thermodynamics and reusability studies†

**DOI:** 10.1039/c9ra06450h

**Published:** 2019-11-20

**Authors:** Dalia Allouss, Younes Essamlali, Othmane Amadine, Achraf Chakir, Mohamed Zahouily

**Affiliations:** Laboratoire de Matériaux, Catalyse et Valorisation des Ressources Naturelles, URAC 24, FST, Université Hassan II-Casablanca Morroco; VARENA Center, MAScIR Foundation, Rabat Design Rue Mohamed El Jazouli, Medinat Al Irfane 10100-Rabat Morroco m.zahouily@mascir.com

## Abstract

Environment-friendly composite hydrogel beads based on carboxymethyl cellulose (CMC), alginate (Alg) and graphene oxide (GO) were synthesized by an ionotropic gelation technique and studied as an efficient adsorbent for methylene blue (MB). The chemical structure and surface morphology of the prepared hydrogel beads were characterized by Fourier transform infrared spectroscopy (FTIR), scanning electron microscopy (SEM), thermogravimetric analysis (TGA), differential thermal analysis (DTA) and point of zero charge (pH_pzc_). A hybrid response surface methodology integrated Box–Behnken design (RSM-BBD) was successfully developed to model, simulate, and optimize the biosorption process. The synergistic effects between three critical independent variables including adsorbent dose (0.3–0.7 g), pH of the MB solution (6.5–9.5) and initial MB concentration (15–45 mg L^−1^) on the MB adsorption capacity (mg g^−1^) and removal efficiency (%) were statistically studied and optimized. The performance of the RSM-BBD method was found to be very impressive and efficient. Results proved that the adsorption process follows a polynomial quadratic model since high regression parameters were obtained (*R*^2^-value = 99.8% and adjusted *R*^2^-value = 99.3%). Analysis of variance (ANOVA) further confirms the validity of the suggested model. The optimal conditions for 96.22 ± 2.96% MB removal were predicted to be 0.6 g of CMC-Alg/GO hydrogel beads, MB concentration of 15 mg L^−1^ and pH of 9.5 within 120 min. The adsorption equilibrium is better described by the Freundlich isotherm, indicating that physisorption is the rate controlling mechanism. The MB adsorption process was thermodynamically spontaneous and endothermic. A reusability study revealed that the prepared adsorbent is readily reusable. The adsorbent still maintains its ability to adsorb MB for up to four cycles. Results reported in this study demonstrated that CMC-Alg/GO hydrogel beads are an effective, promising and recyclable adsorbent for the removal of MB from aqueous solutions.

## Abstract 2

Environment-friendly composite hydrogel beads based on carboxymethyl cellulose (CMC), alginate (Alg) and graphene oxide (GO) were synthesized by an ionotropic gelation technique and studied as an efficient adsorbent for methylene blue (MB).

## Graphical abstract

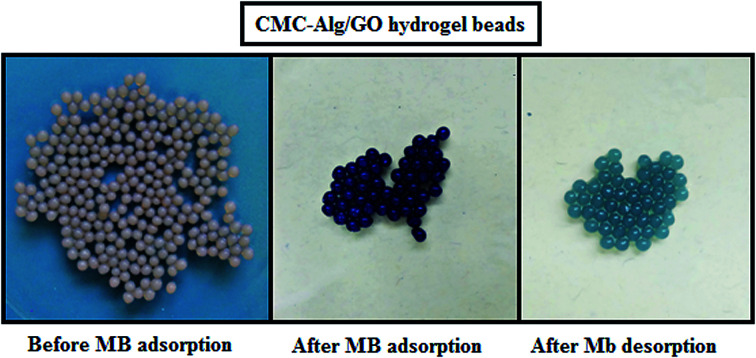

## 1. Introduction

The effluents charged with organic dyes are considered a serious issue due to their negative impact on the aquatic environment and other living species. Currently, many industries such as textile, paper, rubber, plastics, leather, cosmetic, pharmaceutical, and food use natural or synthetic dyes in their processes and apply them to their final products, which results in the generation of huge amounts of wastewater.^1^ The discharge of these highly toxic effluents into the aquatic environment brings about serious issues by causing the reduction of light penetration and photosynthesis. Furthermore, most organic dyes contained in wastewater, particularly the synthetic ones, could decompose into carcinogenic aromatic molecules, which could cause serious health problems to both humans and animals.^2^ Methylene blue (MB), one of the most thiazine dyes widely used in the textile industry for cotton dying, is considered very toxic dye. Several studies highlighted the adverse effects of MB such as eye injury, nausea, vomiting, *etc.*^3^ Consequently, wastewater treatment to remove dyes is one of the biggest challenges that both industrial and academics are facing today. Currently, several physical, chemical, and biological methods such as biological treatment, coagulation/flocculation, membrane filtration, photo-catalysis and adsorption have been investigated for the removal of dyes from aqueous solutions.^4^ The efficiency of each method predominantly depends on the type of dye to be removed, its composition and concentration. Among the above-mentioned techniques, adsorption is recognized as the most suitable and promising technique owing to its simplicity, effectiveness, easy operation, and excellent adsorption ability to remove various harmful compounds from contaminated effluents. The efficiency of the adsorption process depend on different factors including the nature of the adsorbent, the origin of the dyes, and the type of the interactions that may occurred between the adsorbent and the adsorbate.^5^ Over the last decades, various natural and synthetic adsorbents have been developed for the removal of dyes from aqueous solutions. However, most of these adsorbents were not widely used at large scale because of either economic or technological considerations. Thus, there is impelling needs for the development of new low-cost and effective adsorbents, such those derived from natural materials. For instance, polysaccharide-based materials have been extensively used and investigated as adsorbents in most of the adsorption processes owing to their effectiveness, sustainability, and adsorption ability.^6^ These biopolymers show an interesting and attractive alternative as adsorbents for the adsorption of organic dyes because of their abundant functional groups, chemical stability, hydrophilicity and renewability.^7^ Typical examples of biopolymers used for the removal of methylene blue from aqueous solutions include carboxymethyl cellulose,^8^ immobilized microcrystalline cellulose,^9^ chitosan/sepiolite composite,^10^ chitin,^11^ magnetic carboxymethyl cellulose microspheres,^12^ polyacrylamide-sodium alginate microspheres,^13^ starch,^14^ graphene oxide/calcium alginate composites^15^ and activated biomass/alginate beads.^16^

To date, graphene oxide (GO), the oxidized form of graphite,^17^ has been widely and intensively used as a promising adsorbent for the removal of organic molecules from aqueous solutions. Its unique physicochemical and morphological properties such as large surface area, abundant oxygen-containing functional groups^18^ enable it to be used as an alternative adsorbent for dyes and heavy metals.^15^ However, the use of GO in powder form is very limited because of its high dispersibility in water, which restrict its recovery and reusability. One of the most effective approaches to overcome this issue was to immobilize GO nanosheets with other biopolymer for the design of new hybrid GO-based materials. Li *et al.* reported the effective use of calcium alginate/GO composites for the removal of MB from aqueous solutions. They reported a removal efficiency of about 92.7% at pH of 10.^15^ In addition, Liu *et al.* have explored the adsorption capacity of carboxymethyl cellulose, K-carrageen and activated montmorillonite composite beads for the removal of MB. These authors found that the removal efficiency of MB could reach 92% within 120 min.^19^

On the other hand, the adsorption process involves the interaction of large number of operating variables in a non-linear way. In this case, the conventional and classical method for adsorption optimisation is no longer effective since it requires large number of experimental runs and is also time consuming. In addition, this method does not describe the interaction effects of all the operating factors involved in adsorption process. To overcome these limitations, researchers resort statistical experimental design such as response surface methodology (RSM) for effective optimization of the adsorption process. RSM is considered as a powerful mathematical and statistical tool for designing experiments, establishing models by studying the effect of several operating variables by varying them simultaneously.^20^ The main objective of RSM is to get the optimum working conditions in a short time with a limited number of experiments.^21^ Among the various matrix designs, Box–Behnken design (BBD) recommended a three-level incomplete factorial designs. The total experiments is diminished in a quadratic model fitting and it is excelling to adopt second-order polynomial model to precisely express linear interactions and quadratic effects.^22^ For instance, M. Cobas *et al.* reported the effectiveness of the RSM-BBD as a statistical technique to improve and optimize the adsorption process of leather dyes effluents by F. Vesiculosus. Three different factors were considered in the optimisation process namely salt effect, pH and bio-sorbent dosage.^22^

To the best of our knowledge, no study has dealt with precise RSM-BBD based optimization for the study of the removal of MB from aqueous solution over CMC-Alg/GO hydrogel beads. In this work, we report on the use of RSM to design the experimental runs, to develop more accurate model and to optimize different process variables. The adsorption process was optimized by varying three process variables namely adsorbent dosage, solution pH and initial MB concentration. Besides, study of adsorption isotherms, kinetics, thermodynamics, and adsorbent regeneration were also carried out to highlight the adsorption mechanism.

## 2. Experimental section

### 2.1. Materials and apparatus

Sodium alginate (Na-Alg) and carboxymethyl cellulose (CMC) were purchased from Sigma Aldrich and were used as purchased. Aluminium chloride (AlCl_3_, 6H_2_O, 99.3%) was supplied by VWR international PROLABO. Methylene blue (MB), Hydrochloric acid (HCl) and sodium hydroxide (NaOH) were provided from Sigma Aldrich and were used as received. Graphite powder (99.99%) with an average particle size ≤ 20 μm was supplied from VWR International. Graphene oxide (GO) was prepared by graphite powder following a modified Hammers method as reported in our earlier work.^17^ Distilled water (DW) was employed throughout the experiments. The determination of the point of zero charge was carried out to investigate the surface charge of wet CMC-Alg/GO gel beads by the method depicted elsewhere.^23^ Typically, about 300 mg of wet prepared beads were added to 50 mL of 0.1 M NaNO_3_. The pH values of each solution were adjusted from 2 to 11, using either 0.01 M HCl or 0.01 M NaOH. The solutions were maintained under gentle stirring for 24 h and then the final pH values were measured. The pH_pzc_ was defined as the point of intersection of the plot of ΔpH (ΔpH = pH_f_ − pH_i_) *vs.* pH_i_ and the *X*-axis. The FTIR spectra of the raw materials and dry CMC-Alg/GO beads before and after MB adsorption were recorded on a SHIMADZU (IRAffinity-1S) spectrometer using ATR technique which was done between 500 and 4000 cm^−1^. The surface morphologies of the prepared adsorbent were determined using scanning electron microscope (SEM) analysis (Tecnai G2 microscope at 120 kV). Samples were dried at 60 °C until constant weight before being analyzed. Thermo-gravimetric analysis (TGA) and Differential Thermal Analysis (DTA) were conducted under air in a TA Instrument Q500 apparatus at a heating rate of 10 °C min^−1^ between 25 and 600 °C.

### 2.2. Preparation of CMC-Alg/GO hydrogel beads

**Fig. 1:**
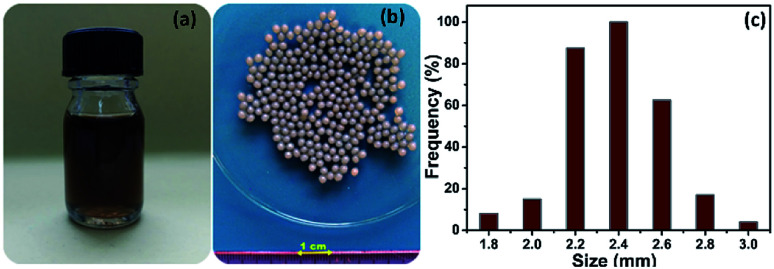
Digital images of (a) CMC-Alg/GO solution (b) wet CMC-Alg/GO hydrogel beads and (c) size distribution (average of 60 beads).

A well-defined amount of carboxymethyl cellulose (CMC) was dissolved in 100 mL of distilled water (1% w/v) under strong magnetic stirring for 1 h at room temperature. Then, 1 g of sodium alginate was added into the CMC solution under magnetic stirring to produce a CMC-Alg homogeneous transparent mixed solution. GO powder (20 mg) was separately dispersed into 20 mL distilled water and sonicated using digital ultrasonic bath (POWER SONIC420, 40 kHz) for 1 h. The aqueous GO solution was then slowly added to the CMC-Alg solution under vigorous magnetic stirring until complete homogenization to make the CMC-Alg/GO solution (Fig. 1a). This solution was then gradually dripped through a 0.8 mm needle into a magnetically stirred aqueous coagulation bath of AlCl_3_, 6H_2_O (1.0 M, 150 mL) at room temperature (25 °C). The obtained gel beads were then left in the coagulation bath for 24 h without any stirring for complete cross-linking reaction between free carboxylic groups of CMC and Al^3+^ ions. Finally, the CMC-Alg/GO gel beads were repeatedly washed with distilled water. The obtained hydrogel beads (Fig. 1b) are spherical with an average size of 2.4 ± 0.2 mm (Fig. 1c). It should be noted that the CMC-Alg/GO beads were kept on a wet form for further utilization.

### 2.3. Process variables

In adsorption processes, the traditional one factor at time optimization for achieving maximum dye removal requires several experiences and it is time consuming. Recently, response surface methodology (RSM) has been demonstrated as beneficial technique to reduce both time and costs of experimentation.^20^ This methodology is herein adopted for the optimization of process parameters for MB removal from aqueous solutions. In this research paper, a three-level factorial Box–Behnken design was applied with 15 batch adsorption experiments to optimize the removal efficiency (%) and adsorption capacity (mg g^−1^) of MB using CMC-Alg/GO hydrogel beads as adsorbent. The three independent variables including adsorbent dose (g), pH of MB solution and initial MB concentration (mg L^−1^) were selected to investigate their linear effects, quadratic effects and double interactions between them. These parameters were chosen based on literature reports for dye adsorption as well as on the results of preliminary studies performed in the laboratory. The other parameters namely the contact time and temperature were fixed at 120 min and 25 °C, respectively. In this study, the ANOVA and 2D–3D surface plots were calculated using Nemrodw software. The levels of variables to be optimized considered for the adsorption of MB onto CMC-Alg/GO beads are presented in Table 1.

**Table:** Experimental range and levels in the BBD

Factors	Levels		
	Low (−1)	Center (0)	High (+1)
*X*_1_: adsorbent dose (g)	0.3	0.5	0.7
*X*_2_: pH	6.5	8	9.5
*X*_3_: initial MB concentration (mg L^−1^)	15	30	45

### 2.4. Batch adsorption experiments

Batch adsorption experiments were conducted in glass beakers containing 25 mL of MB solution of known concentration (15 mg L^−1^) to which appropriate weight of wet CMC-Alg/GO bioadsorbent was added. The beakers were stirred at a constant speed of 150 rpm in an orbital shaker for different time intervals ranging from 10 to 260 min. Operating parameters such as contact time, initial MB concentration, and temperature were studied and optimized for maximum MB removal based on RSM-BBD methodology. Upon experiment completion, the residual concentration of MB was measured using a UV-Vis spectrophotometer (JENWAY 6715) at a wavelength of 664 nm which corresponds to the maximum absorbance of MB dye using a calibration curve. Thermodynamic study was carried out by varying adsorption temperature from 25 to 55 °C at fixed adsorbent dose and MB concentration. The adsorption experiments were carried out in triplicate and average values were used for data analysis. The MB removal efficiency (%) and adsorption capacity (*q*_*t*_, mg g^−1^) were determined by using the following equations:^4^1
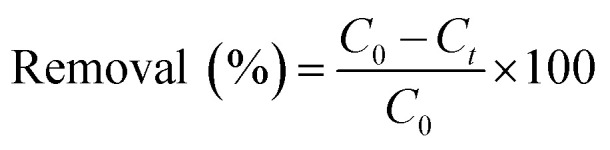
2
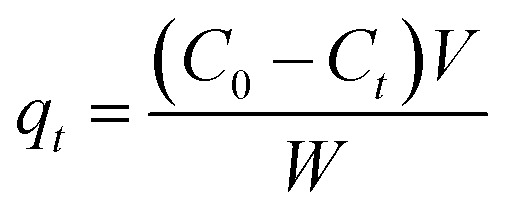
where *C*_0_ is the initial dye concentration (mg L^−1^), *C*_e_ is the dye concentration at equilibrium (mg L^−1^), *C*_*t*_ is the dye concentration at time *t* (mg L^−1^), and *V* is the volume of the dye solution (L) and *W* is the weight of dry beads (g).

### 2.5. Desorption and regeneration study

The recovery and reusability of a solid adsorbent is one of the most important features in practical applications for dye removal from wastewater.^24^ Batch mode was used for four cycles of MB adsorption/desorption study. 0.6 g of wet hydrogel beads was stirred in 50 mL of aqueous MB solution (15 mg L^−1^) for 120 min. After equilibrium, the desorption of the as-prepared adsorbent was carried out by introducing 0.6 g of the saturated CMC-Alg/GO into 50 mL of HCl at 0.5 M as a desorbing agent. The mixture was stirred in a rotary shaker for 60 min. The recycled adsorbent was then filtered, washed thoroughly with distilled water, and finally stored in distilled water until being used. The change of colour after MB adsorption and desorption was exposed in Fig. S1 (ESI).†

## 3. Results and discussions

### 3.1. Characterization of CMC-Alg/GO beads

**Fig. 2:**
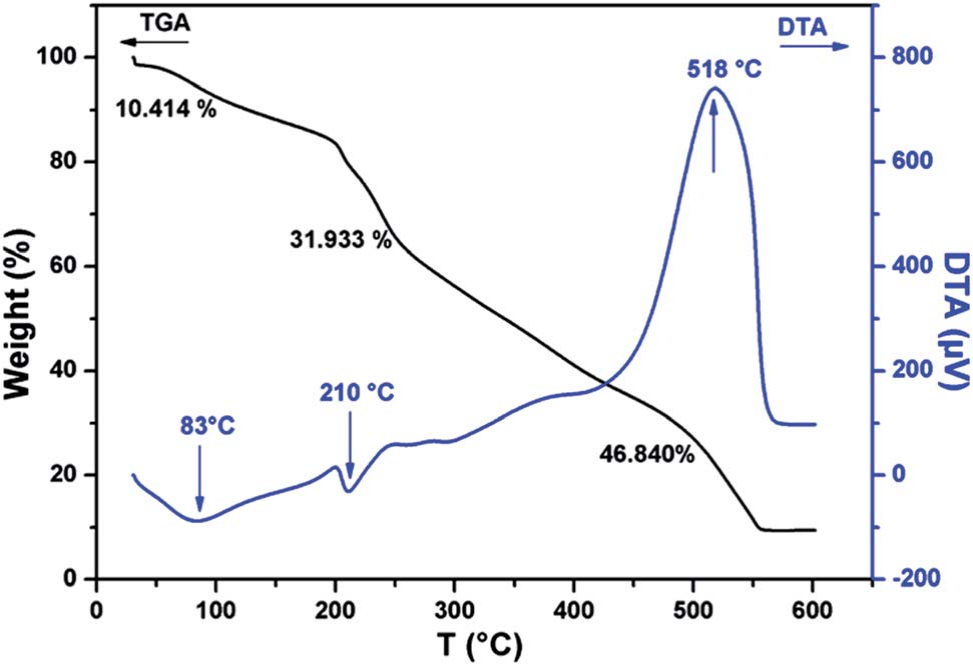
TGA and DTA curves of dry CMC-Alg/GO beads.

The thermal stability of CMC-Alg/GO beads was investigated by TGA/DTA and the corresponding curves are given in Fig. 2. TGA curve of CMC-Alg/GO beads showed three distinct weight losses corresponding to three DTA peaks. The first weight loss of about 10.41% occurred at temperature below 150 °C was mainly due to the evaporation of physisorbed water and the retained moisture. This weight loss leads to the appearance of an endothermic DTA peak at approximately 83 °C. The second weight loss beginning at approximately 155 °C and ending at about 440 °C was mainly due to the thermal decomposition of oxygen-containing functional groups of GO and the depolymerization^25^ of the CMC-Alg blend. This process is followed with an endothermic DTA peak with maximum at 210 °C. The last weight loss occurring between 450 to 550 °C was assigned to the rupture of biopolymers chains and cleavage of the C–C bonds. At this stage, complete decomposition of the carbonaceous into carbonate as a by-product. This last process was followed with exothermic peaks with maximum at 518 °C and with the weight loss of about 46.84%. The exothermic DTA peak at 518 °C was related to the energy released from burning organic compounds resulting from the thermal degradation of CMC-Alg/GO.

**Fig. 3:**
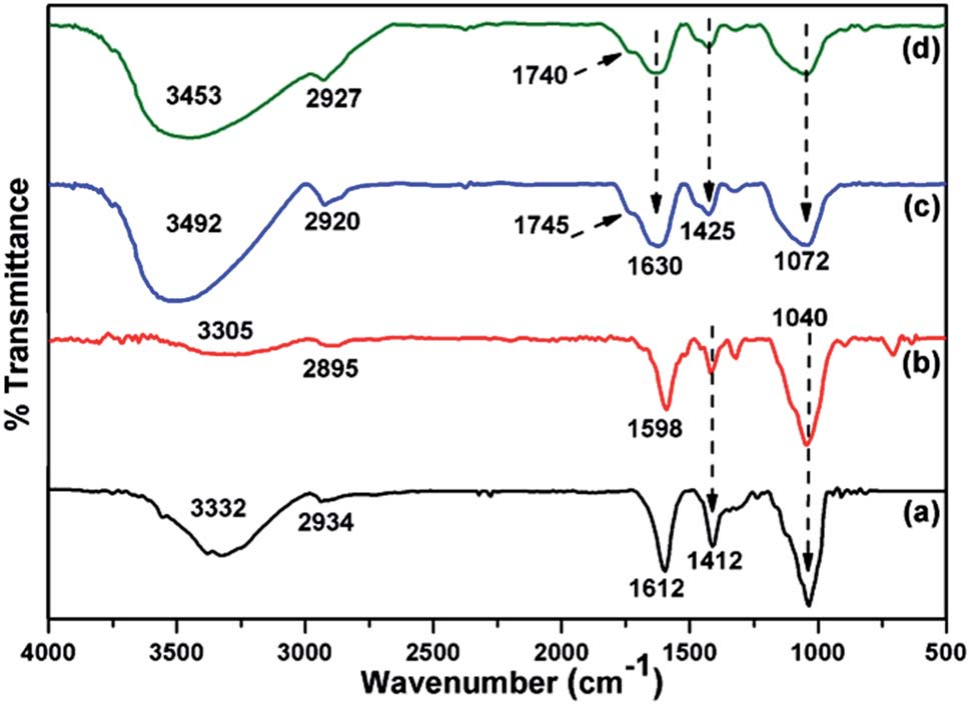
ATR-FTIR spectra of (a) SA powder, (b) CMC powder, (c) CMC-Alg beads and (d) CMC-Alg/GO beads.

FTIR was used to study the chemical structure of the CMC-Alg/GO beads and the corresponding spectra are shown in Fig. 3. The absorption band of sodium alginate (Alg) powder appearing at 2934 cm^−1^ was assigned to C–H stretching and those observed at 1612 and 1412 cm^−1^ were assigned to the symmetric and asymmetric COO^−^ stretching vibrations of the free carboxylate groups (Fig. 3a). There are two further strong bands at 3332 cm^−1^ and 1040 due to O–H and C–O–C stretching vibrations of alcohol and ethers, respectively (Fig. 3a). For carboxymethyl cellulose (CMC) powder, vibrations from CMC homopolymeric are clearly evident. The absorption bands observed at 3305, 2895, 1598 and 1412 cm^−1^ can be attributed to O–H stretching (hydroxyl), C–H stretching, asymmetric and symmetric stretching of carboxylic groups (Fig. 3b). Furthermore, the distinct FTIR absorption bands observed at 1322 and 1040 cm^−1^ can be assigned to C–O stretching and C–O–C stretching of the saccharide structure in anhydroglucose unit.^26^ For the Al^3+^ cross-linked CMC/SA beads, the stretching vibration of the OH group (3305 cm^−1^) and COO^−^ group (1040 cm^−1^) in CMC were shifted to 3492 and 1072 cm^−1^, respectively. Moreover, the asymmetric and symmetric stretching vibrations of carboxylic groups observed at 1598 and 1412 cm^−1^ were also shifted to 1630 and 1425 cm^−1^, respectively. These changes were related to the hydrogen bonding-type interaction between CMC and Alg and to the cross-linking reaction between Al^3+^ and carboxylic groups of CMC, which was further evidenced by the presence of a small peak at 1745 and 1740 cm^−1^ for CMC-Alg gel beads.^12^ After loading the CMC/Alg matrix with 1 wt% GO, the bands at 2920 and 3492 cm^−1^ in CMC/Alg were shifted to 2927 and 3453 cm^−1^, respectively, while the other bands remained almost unchanged (Fig. 3d). Such shifting confirms that GO acts as a physical cross-linking agent to yield hydrogen bonding type interactions, which can in turn promote miscibility and compatibility between CMC and SA chains.^27^

The FTIR spectra of CMC-Alg/GO before and after MB adsorption are shown in Fig. S2 (ESI).† After MB adsorption, two new and small absorption bands appears at about 1246 and 736 cm^−1^, which were assigned to C–N stretching and aromatic C–H of out plane bending of MB, respectively.^12^ The presence of blue shifting from 3453 to 3736 cm^−1^ suggested performed the involvement of hydrogen bonding between MB and CMC-Alg/GO beads. This displacement could probably also be due to the interaction between cationic MB and the negatively charged surface of CMC-Alg/GO beads. In addition, in as much as presence of the blue shifting from 1630 to 1742 cm^−1^ suggested that involvement the participation of carboxylic groups of SA are also involved in during the adsorption process.

**Fig. 4:**
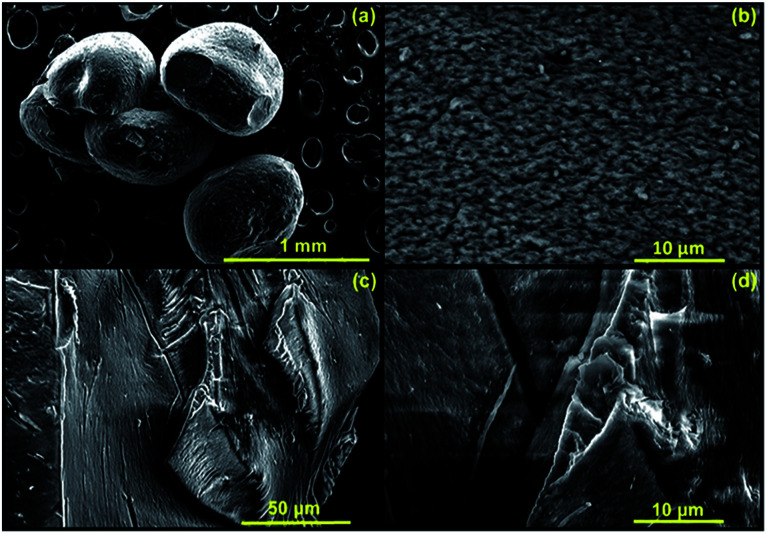
SEM images of (a) dry CMC-Alg/GO bead, (b) surface of CMC-Alg/GO bead and (c and d) cross-section of CMC-Alg/GO bead.

As a part of a morphological analysis, surface morphology of the dried CMC-Alg/GO adsorbent was investigated by SEM analysis (Fig. 4). Surface morphology analysis revealed that the CMC-Alg/GO sample exhibited a condensed heterogeneous microstructure with clear surface roughness (Fig. 4a and b). The surface is very dense and compact without any visible porosity. This could be due the dramatic shrinking and contraction of the CMC-Alg/GO beads during the evaporative drying which result in the formation of a compact structure with virtually no porous structure (Fig. 4b). Furthermore, cross-sectional SEM images (Fig. 4c and d) revealed that the sample is very rough with plenty of wrinkles and folds. Similar results were obtained by Zhang *et al.* when using magnetic bentonite/carboxymethyl chitosan/sodium alginate hydrogel beads for Cu(ii) ions removal from aqueous solution.^25^

It is well known that the ionization state of adsorbate and the surface charge of the adsorbent are pH dependent. For this reasons, investigation of the point of zero charge (pH_pzc_) value is great importance as it gives information on pH ranges where the surface charge of the adsorbent is positively or negatively charged.^28^ The pH_pzc_ of the CMC-Alg/GO beads was determined and was found to be 3.5 as shown in Fig. S3 (ESI).† This indicates that for pH > pH_pzc_ = 3.5, the surface charge of the CMC-Alg/GO adsorbent is negatively charged, there by suitable for cationic dye removal. In this case, the uptake of MB may be ascribed to the attraction electrostatic forces between the negatively charged surface (deprotonation of hydroxyl and carboxyl groups) and the positively charged dye. However, at pH value below the pH_pzc_ (pH < 3.5), the adsorbent surface charge becomes positively charged in the studied pH range and thus not suitable for MB removal through electrostatic interactions.

### 3.2. Response surface methodology modelling using Box–Behnken design (BBD)

In this part of work, Box–Behnken design (BBD) coupled with response surface methodology (RSM) were applied to find out the optimum conditions for the removal of MB onto CMC-Alg/GO hydrogel beads. The complete experimental design matrix for three investigated variables together with the actual and predicted responses (*Y*_1_ for the removal efficiency and *Y*_2_ for the adsorption capacity) are given in Table 2.

**Table:** Design matrix for three coded variables together with the actual and predicted responses^a^

Runs	Coded values of the variables						
	*X*_1_	*X*_2_	*X*_3_	*Y*_1_	*Ŷ*_1_	*Y*_2_	*Ŷ*_2_
1	−1.00	−1.00	0.00	51.300	51.200	44.80	46.462
2	1.00	−1.00	0.00	65.300	65.200	24.50	26.163
3	−1.00	1.00	0.00	72.700	72.800	63.40	61.737
4	1.00	1.00	0.00	77.000	77.100	28.90	27.238
5	−1.00	0.00	−1.00	44.300	44.300	10.60	10.600
6	1.00	0.00	−1.00	50.500	50.500	6.50	6.500
7	−1.00	0.00	1.00	45.500	—	88.40	—
8	1.00	0.00	1.00	60.600	—	38.60	—
9	0.00	−1.00	−1.00	57.900	58.000	15.20	13.537
10	0.00	1.00	−1.00	94.800	94.700	24.90	26.562
11	0.00	−1.00	1.00	70.500	70.600	53.10	51.438
12	0.00	1.00	1.00	67.500	67.400	53.10	54.763
13	0.00	0.00	0.00	70.000	71.600	36.90	39.300
14	0.00	0.00	0.00	72.400	71.600	40.50	39.300
15	0.00	0.00	0.00	72.400	71.600	40.50	39.300

a *X*_1_: adsorbent dose (g), *X*_2_: pH, *X*_3_: initial concentration (mg L^−1^), *Y*_1_: removal efficiency (%) and *Y*_2_: adsorption capacity (mg g^−1^).

In response surface methodology (RSM), the most widely used empirical model that describes the relationship between the design variables and the response can be represented by the following second-order polynomial equation:

3 *Ŷ* = *b*_0_ + ∑*b*_*i*_*X*_*i*_ + ∑*b*_*ii*_*X*_*i*_^2^ + ∑*b*_*ij*_*X*_*i*_*X*_*j*_ + *ε*

where *Ŷ* is the predicted response, *b*_0_, *b*_*i*_, *b*_*ii*_, *b*_*ij*_ are regression coefficients for the intercept, linear, quadratic and interactions among input factors, respectively. *X*_*i*_, *X*_*i*_^2^, *X*_*j*_ are levels of independent factors in coded units and *ε* is the error of the model.

The final Box–Behnken design obtained for percentage removal of MB with significant terms was quadratic. The mathematical quadratic polynomial model that correlates the response to the three chosen variables involved in the current adsorption process suggested by the software can be written as:

4 *Ŷ*_1_ = 71.6 + 4.575*X*_1_ + 8.375*X*_2_ − 3.675*X*_3_ − 16.987*X*_1_^2^ + 11.962*X*_2_^2^ − 10.88*X*_3_^2^ − 2.425*X*_1_*X*_2_ − 9.975*X*

The above-mentioned equation (eqn (4)) discloses how the quadratic or interactive model terms affected MB removal from aqueous solution using CMC/Alg-GO as an adsorbent. The adequacy of the established mathematical model in predicting the MB removal efficiency (%) was checked by calculating the statistical parameters like the coefficient of determination (*R*^2^) and the analysis of variance (ANOVA). The ANOVA results of MB removal using CMC/Alg-GO hydrogel beads are given in Table 3.

Analysis of variance (ANOVA) for MB removal efficiency (%) onto CMC/Alg-GO adsorbent beads

**Table:** 

Source	Sum of squares	*Df*	Mean square	*F*-Value	*P*-Value (%)	Comments
Regression	2099.47	9	233.275	187.53	0.0622	SD = 1.14%
Residuals	3.92	3	1.31	—	—	CV = 1.6%
Lack of fit	0.08	1	0.08	0.0417	85.7	*R* ^2^ = 0.998
Pure error	3.84	2	1.92	—	—	*R* ^2^adj = 0.993

**Table:** 

Terms	Coefficient	SE coefficient	*t*-Value	*P*-Value (%)
Constant	71.600	0.659	108.490	0.01
*X*_1_	4.575	0.571	8.000	0.407
*X*_2_	8.375	0.404	20.720	0.0246
*X*_3_	−3.675	0.571	−6.430	0.763
*X*_21_	−16.987	0.719	−23.620	0.0166
*X*_22_	11.962	0.719	16.630	0.0473
*X*_23_	−10.887	0.719	−15.140	0.0626
*X*_1_ *X*_2_	−2.425	0.571	−4.240	2.40
*X*_1_ *X*_3_	1.475	0.989	1.490	23.3
*X*_2_ *X*_3_	−9.975	0.571	−17.450	0.0410

According to the data in Table 3, it can be clearly seen that the predicted *R*^2^ value (0.998) and adjusted *R*^2^ (0.993) values are closely related to each other and thus are in ideal agreement for the quadratic model. This suggests that the quadratic polynomial model provides an excellent explanation for the relationship between the process variables and the response. The *R*^2^ value of 0.993 implies that 99.3% of the total variation on MB adsorption data can be described by the proposed mathematical model and that only 0.7% of the total variation cannot be described by it. Based on ANOVA results (Table 3), the model F-value of 187.53 and *p*-value of 0.0622% imply that the model is statistically significant. Which means that the proposed mathematical model fitted well to the experimental data based on an insignificant lack-of-fit^29^ (Table 3). In addition, the significance of each of the linear terms (*X*_1_, *X*_2_ and *X*_3_), the interaction terms (*X*_1_*X*_2_, *X*_1_*X*_3_ and *X*_2_*X*_3_), and the quadratic terms (*X*_1_^2^ and *X*_2_^2^) on the response were evaluated by *p*-values. The terms having a *p*-value less than 5% are said to be significant. Here the adsorbent dosage (*X*_1_), pH value (*X*_2_), initial dye concentration (*X*_3_) and their quadratic terms (*X*_1_^2^, *X*_2_^2^ and *X*_3_^2^) to which was added two interaction terms (*X*_1_*X*_2_ and *X*_2_*X*_3_) have *p*-value less than 5% and hence selected as exceedingly significant model terms.

The sign before the selected individual, quadratic or double interactions terms (shown in eqn (4)) was used to determine whether each model terms affect positively or negatively on the MB adsorption process. Based on data in Table 3, most significant factors positively affecting the MB adsorption on CMC-Alg/GO hydrogel beads are the linear terms *X*_1_ (adsorbent dosage) and *X*_2_ (pH value) in the tested range. In the other hand, the negative coefficient values of the linear term (*X*_3_), the quadratic terms (*X*_1_^2^ and *X*_3_^2^) and the interaction terms (*X*_1_*X*_2_ and *X*_2_*X*_3_) signify that these factors negatively affect the response (*i.e.*, adsorption percentage decreases).

The outcomes of ANOVA involves that the lack of fit (*F*-value of 0.0417) is not significant relative to the pure error. There is 85.7% chance that a ‘‘lack of fit *F*-value’’ could happen due to noise (Table 3). This means that the phenomenon has been very well explained by our model with a 95% confidence level.

The plot between experimental (actual) and predicted values of MB removal is shown in Fig. S4 (ESI).† From this figure, it is clearly seen that the average differences between the predicted and experimental values are less than 0.1, which indicates that most of the data variation was explained by the regression model. In view of these outcomes, we can reasonably conclude that the proposed mathematical model was appropriate and effective for the analysis and the optimization of MB adsorption by CMC-Alg/GO beads.

**Fig. 5:**
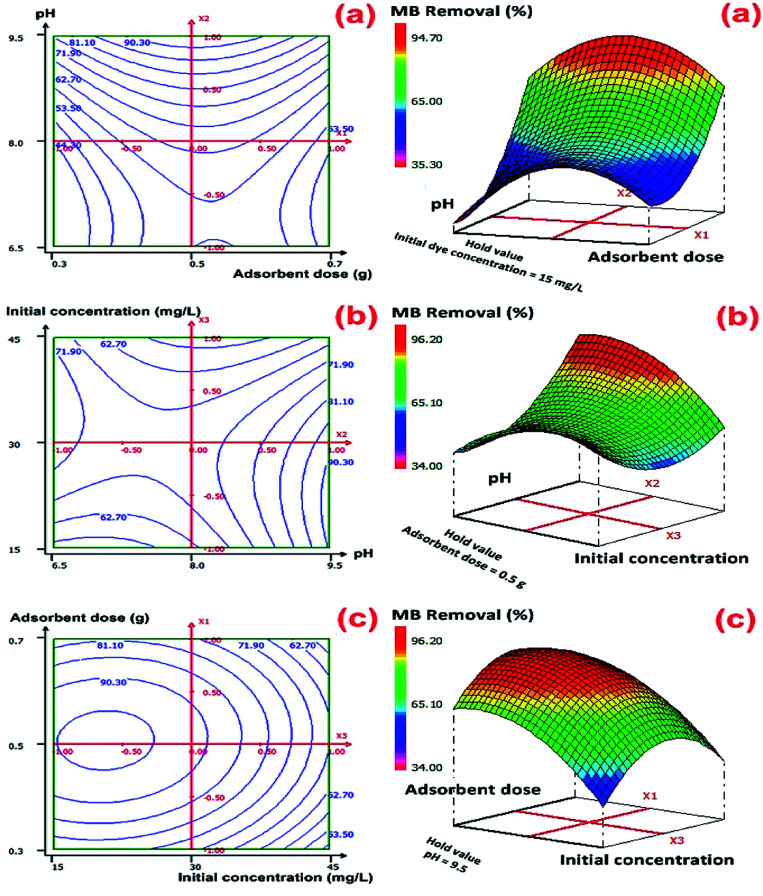
2D–3D surface plots of the effect (a) the adsorbent dose and pH, (b) the initial concentration and pH and (c) the adsorbent dose and initial concentration on MB removal efficiency (%) by CMC-Alg/GO hydrogel beads.

The graphical representations of the regression equation were shown by the 2D-contour and 3D-response surface plots in Fig. 5 in the case of MB removal and Fig. S5 (ESI†) for the adsorption capacity (mg g^−1^).

From these graphical representations, we can clearly deduce on the significance of binary interactions between different selected variables (pH, initial MB concentration, and adsorbent dosage). Fig. 5 shows that the *X*_1_*X*_2_ interaction over optimized condition at a fixed initial MB concentration of 15 mg L^−1^ is the most significant interaction term in predicting maximal value for MB removal. According to these plots, the predicted value of optimum MB removal was found to be (96.22 ± 2.96)% with the use 0.6 g of CMC-Alg/GO hydrogel beads, MB concentration of 15 mg L^−1^ and pH of 9.5 during 120 min of agitation time. With the increasing of initial pH solution, the percentage of MB removal achieved maximal value. The adopted approach was very effective and the results obtained indicated that RSM-BBD method provided high percentage of MB removal values based on several scenarios given by the software.

### 3.3. Adsorption kinetics

**Fig. 6:**
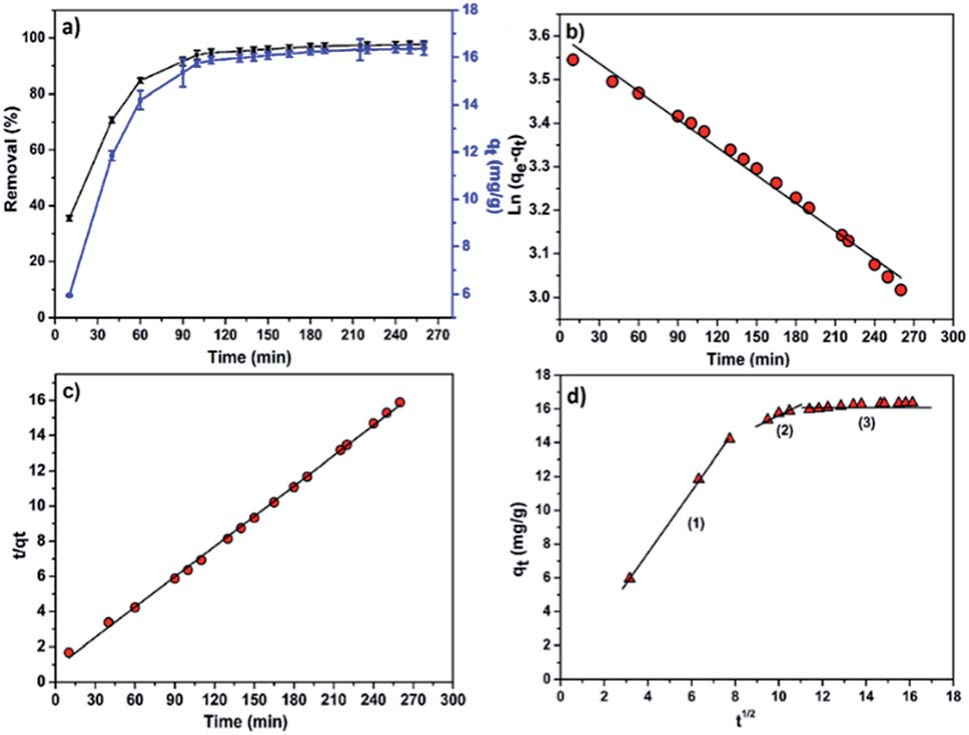
(a) Effect of contact time on the MB removal (%) and adsorption capacity (mg g^−1^), (b) pseudo-first order, (c) pseudo-second order kinetic and (d) intra-particle diffusion kinetic models.

To study the effect of contact time on the efficiency of the adsorption process, batch adsorption experiments were conducted at a fixed adsorbent dose of 0.6 g and initial MB concentration of 15 mg L^−1^ while varying contact time from 10 to 260 min. Samples were collected from the flask at predetermined time intervals for analyzing the residual MB concentration in the solution. Fig. 6a showed the effect of contact time on the adsorption of MB onto CMC-Alg/GO hydrogel beads. According to this figure, it is clear that the removal of MB increased rapidly during the first 60 min followed by a slower MB uptake until 90 min because the adsorption sites got occupied. The equilibrium was achieved almost within 90 min. The maximum removal efficiency of MB by CMC-Alg/GO (91%) over a period of 90 min is quite similar to that of Ball-milled biochar encapsulated in calcium alginate beads (CA-BMB) over a period of 16 h.^30^

The pseudo-1^st^-order, pseudo-2^nd^-order, and intraparticle diffusion models were separately applied to fit our experimental data in an attempt to explain the adsorption kinetic of MB onto CMC-Alg/GO hydrogel beads. The linear equation of pseudo-1^st^-order kinetic model is represented in eqn (5):^30^

5 ln(*q*_e_ − *q*_*t*_) = ln *q*_e_ − *k*_1_*t*

where *k*_1_ is the equilibrium rate constant of pseudo-1^st^-order model (min^−1^), *q*_e_ and *q*_*t*_ refer to the amounts of dye adsorbed on adsorbent (mg g^−1^) at equilibrium and at time *t*, respectively.

The linear plot of ln(*q*_e_ − *q*_*t*_) *versus t* is shown in Fig. 6b and Table 4 lists the values of the rate constant *k*_1_, the predicted *q*_e_ value and the correlation coefficient *R*^2^. Usually the best fit can be selected based on the value of the correlation coefficient (*R*^2^) and predicted *q*_e_ value. In our case, data in Table 4 shows regression coefficient more than 0.99 but the calculated *q*_e_ value did not deviate reasonably from the experimental value. Such results suggest that our experimental data did not fit well the pseudo-1^st^-order model and thus the adsorption process of MB on CMC-Alg/GO beads did not obey the pseudo-1^st^-order model. Therefore, the 2^nd^-order kinetic model was applied to fit our experimental data using the following eqn (6).^30^6
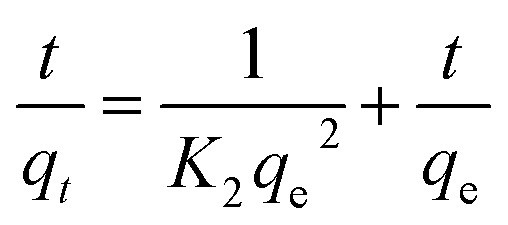
where *q*_e_ and *q*_*t*_ refer to the adsorption capacity (mg g^−1^) at equilibrium and at time (*t*), respectively, *K*_2_ is the equilibrium rate constant of pseudo-2^nd^-order model (g mg^−1^ min^−1^).

**Table:** Kinetic model parameters for MB adsorption on the CMC-Alg/Go hydrogel beads

Models	Parameters	CMC-Alg/GO beads
Pseudo-first order kinetic	*q*_e,exp_ (mg g^−1^)	16.36 ± 1.25
	*K*_1_ (min^−1^)	0.00214 ± 0.00006
	*R* ^2^	0.99445
	*q*_e,calc_ (mg g^−1^)	36.65 ± 0.01
	RMSE	0.01753
Pseudo-second order kinetic	*q*_e,exp_ (mg g^−1^)	16.36 ± 1.25
	*K*_2_ (g mg^−1^ min^−1^)	0.004 ± 0.085
	*R* ^2^	0.99941
	*q*_e,cal_ (mg g^−1^)	17.4307 ± 0.0005
	RMSE	0.15238

The linear plot shown in Fig. 6c was used to estimate the adsorption kinetics parameters of the pseudo-2^nd^-order model and the obtained results are given in Table 4.

Based on data in Table 4, it is clearly observed that the correlation coefficient *R*^2^ for the pseudo second order kinetic model obtained for a concentration of 15 mg L^−1^ is higher than the *R*^2^ for pseudo-first-order kinetic model. Furthermore, the predicted *q*_e_ value (*q*_e,calc_ = 17.43 mg g^−1^) deviated reasonably from the experimental value (*q*_e,exp_ = 16.36 ± 1.25 mg g^−1^) for the pseudo-second-order model compared to that obtained for the pseudo-first-order model (Table 4). Thus, the experimental kinetic data for the adsorption of MB can be more likely described by the pseudo-second-order model in which the efficiency of the adsorption of MB strongly depends on the number of available active sites of the CMC-Alg/GO adsorbent.^31^

Furthermore, the intraparticle diffusion model was also used to identify if the mechanism of intraparticle diffusion is the rate limiting step in the overall adsorption of MB onto CMC-Alg/GO, as expressed in eqn (7).^32^

7 *q*_*t*_ = *k*_i_*t*^1/2^ + *C*_i_

where *k*_i_ is the intraparticle diffusion rate constant (g mg^−1^ min^−1/2^) and *C*_i_ is a constant related to the extent of the boundary layer effect. It provides information about the thickness of the boundary layer and the resistance to the external mass transfer.

In our study, the contribution of the intra-particle diffusion was explored by plotting *q*_*t*_*versus t*^1/2^. Fig. 6d shows the intra-particle diffusion model for MB adsorption onto CMC-Alg/GO hydrogel beads. Based on the visual assessment of the plots in Fig. 6d, it is clearly observed that the value of *q*_*t*_ was linearly correlated with values of *t*^1/2^ in three gradual adsorption stages. This suggests that the adsorption process of MB dye by CMC-Alg/GO hydrogel beads occurred in three distinct stages represented by three distinct linear plots (plots in Fig. 6d). The rate constant *K*_i_ for different stage is directly evaluated from the slope of the regression line (Table 5). The first linear section corresponds to the diffusion of the MB molecules from the bulk solution to the outer surface of the CMC-Alg/GO hydrogel beads, while the second stage represents the intra-particle diffusion in which the MB molecules diffuse through the adsorbent pores to the internal surface of the beads. The third one is the equilibrium stage. The perfect linearity of the plots given in Fig. 6d and the higher values *R*^2^ demonstrated that intra-particle diffusion played a significant role in the adsorption of MB by CMC-Alg/GO hydrogel beads. This suggests that the rate limiting step is the intraparticle diffusion process.

**Table:** Intra-particle diffusion kinetic parameters for the removal of MB onto CMC-Alg/GO beads

Parameters	Step (1)	Step (2)	Step (3)
*K*_1_ (mg g^−1^ min^−1/2^)	1.81 ± 0.05	0.53 ± 0.14	0.084 ± 0.007
*C*_1_ (mg g^−1^)	0.26 ± 0.30	10.32 ± 1.43	15.05 ± 0.10
*R* ^2^	0.999	0.965	0.964
RMSE	0.167	0.102	0.040

### 3.4. Adsorption isotherms

**Fig. 7:**
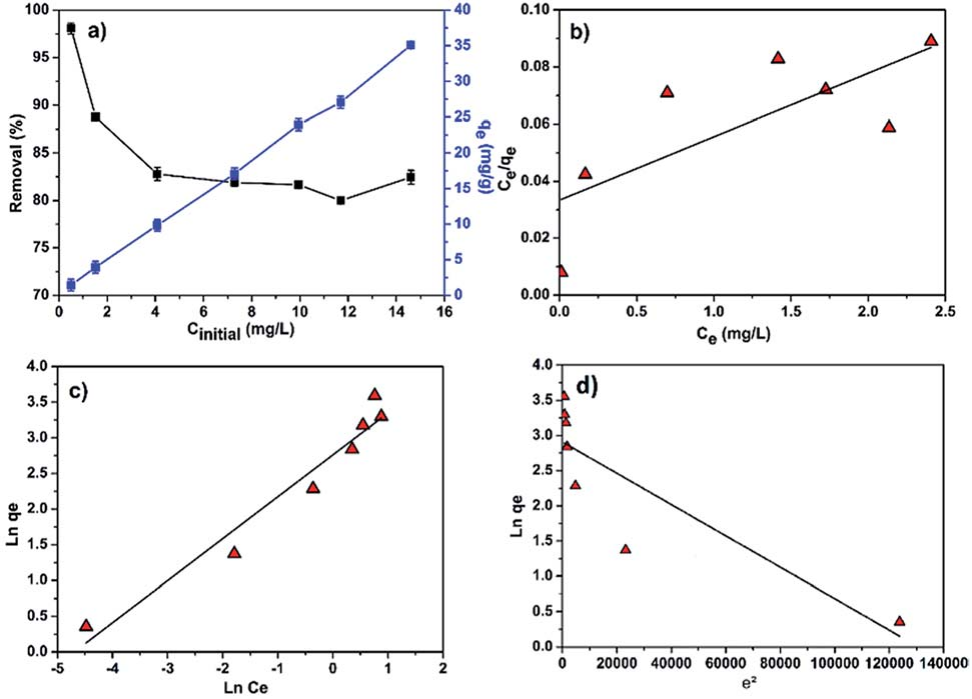
(a) Effect of initial MB concentration on the removal efficiency and adsorption capacity; linear fitting plots of (b) Langmuir (c) Freundlich and (d) Dubinin–Radushkevich isotherms.

To study the effect of initial MB concentration on the efficiency of the adsorption process and to better understand the adsorption patterns, adsorption experiments were carried out by varying the initial concentration of MB from 0.5 to 15 mg L^−1^. In such experiment, the adsorbent dose and the contact time were maintained at 0.35 g L^−1^ (*i.e.* 0.6 g of adsorbent) and 120 min, respectively. The effect of MB concentration on the removal efficiency (%) and adsorption capacity (mg g^−1^) at the above operating conditions is depicted in Fig. 7a.

According to the Fig. 7a, it is clearly observed that the adsorption capacity increases from 1.5 to 35 mg g^−1^ with increasing MB concentration from 0.5 to 15 mg L^−1^ while the percentage of MB removal evidently decreased with increasing initial dye concentration. A higher MB concentration leads to higher driving force for mass transfer from the bulk solution to the adsorbent surface, resulting in a faster sorbent uptake and higher adsorption capacity. Additionally, as the MB concentration increases, adsorption sites got occupied which results in the decrease in the removal efficiency as shown in Fig. 7a.

Different models, namely, Langmuir, Freundlich and Dubinin–Radushkevich (D–R) isotherms were used to fit our experimental data for proper description of nature of the interaction between the MB and CMC-Alg/GO adsorbent at equilibrium. These models help also to understand the mechanism of adsorption and provide some insight into the distribution of available adsorption sites across the adsorbent surface.^33^

Eqn (8), (9) and (10) describe the linear form of the Langmuir, Freundlich, and Dubinin–Radushkevich isotherms, respectively:8
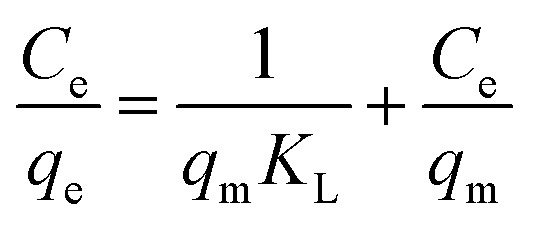
9
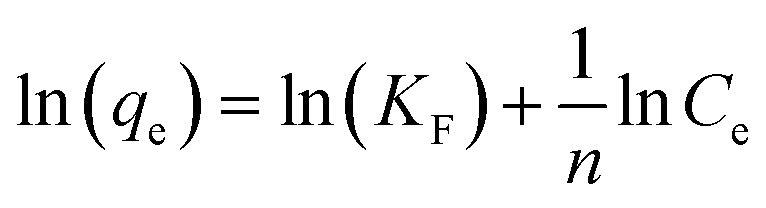

10 *ln*(*q*_e_) = ln(*q*_m_) − *βε*^2^, where *ε* = *RT* ln(1 + 1/*C*_e_)

where *q*_e_ is the adsorption capacity at equilibrium (mg g^−1^), *C*_e_ is the MB concentration at equilibrium (mg L^−1^), *K*_L_ is the Langmuir adsorption constant (L mg^−1^), *q*_m_ is the maximum adsorption capacity (mg g^−1^), *K*_F_ is Freundlich adsorption constant, *n* is the heterogeneity factor and *β* is a constant related to the adsorption free energy (mol^2^ kJ^−2^).

The linear fitting of the Langmuir and Freundlich models to the equilibrium data is displayed in Fig. 7b and c, respectively. Table 6 summarizes the Langmuir and Freundlich experimental constants. Based on the visual assessment of the plots in Fig. 7b and c and according to the high correlation coefficient (*R*^2^), we can reasonably conclude that the isotherm data were better described by the Freundlich model when using CMC-Alg/GO as an adsorbent. This confirms that the adsorption of MB onto CMC-Alg/GO hydrogel beads involves a multilayer adsorption process on heterogeneous surface. Additionally, the value of 1/*n*, referring to the adsorption intensity of dye onto the adsorbent, was below 1 suggesting that the MB is favorably adsorbed by CMC-Alg/GO.^34^

**Table:** Isotherm parameters for MB adsorption on the CMC-Alg/GO hydrogel beads

Models	Parameters	CMC-Alg/GO beads
Langmuir isotherm	*q*_max_ (mg g^−1^)	45.045 ± 0.019
	*K*_L_ (L mg^−1^)	0.665
	*R*_L_	0.09 to 0.75
	*R* ^2^	0.756
	RMSE	0.0199
Freundlich isotherm	*K*_F_	7.505
	1/*n*	0.587
	*R* ^2^	0.975
	RMSE	0.28622
Dubinin–Radushkevich	*q*_max_ (mg g^−1^)	18.315 ± 0.273
	*β* (mol^2^ kJ^−2^)	0.0224
	*E*_a_ (kJ mol^−1^)	4.724
	*R* ^2^	0.867
	RMSE	0.638

To determine whether the adsorption is a chemical or physical process, Dubinin–Radushkevich (D–R) isotherm model has been applied to fit our experimental adsorption data. D–R isotherm constants namely the adsorption free energy and the maximum adsorption quantity calculated from this model according to eqn (10) are listed in Table 6. Based on the value D–R isotherm constant (*β*) the mean free energy of adsorption (*E*_a_) was calculated using the following eqn (11):11
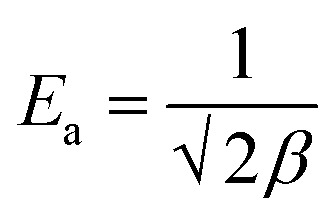


According to the literature, a value of free energy (*E*_a_) comprises between 8 and 16 kJ mol^−1^ indicates that the rate limiting step is chemisorption, while a value less than 8 kJ mol^−1^ was appropriate for physisorption mechanism.^35^ In our study, the value of *E*_a_ was found to be less than 8 kJ mol^−1^ (Table 6), suggesting that the MB adsorption on CMC-Alg/GO beads may be due to physical binding. This leads to the conclusion that the MB binding to the CMC-Alg/GO beads is primarily based on physical processes rather than chemical processes.

### 3.5. Thermodynamic study

Thermodynamic study is of utmost importance for proper prediction of adsorption mechanism. The effect of temperature on the MB removal by CMC-Alg/GO hydrogel beads was explored over the range of 25–55 °C under optimized conditions. The thermodynamic parameters of MB adsorption onto CMC-Alg/GO beads including Gibbs free energy (Δ*G*°), enthalpy (Δ*H*°) and entropy (Δ*S*°), were graphically determined according to the thermodynamic laws through the Gibbs free energy and Van't Hoff equations shown below:^24^

12 Δ*G*° = −*RT* ln *K*

13 Δ*G*° = Δ*H* − *T*Δ*S*°

14
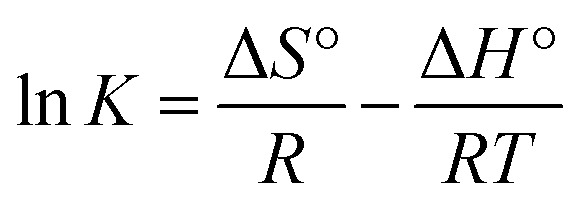
where *K* is the equilibrium constant, *R* is the gas constant (8.314 J mol^−1^ K^−1^) and *T* is the absolute temperature (K).

**Fig. 8:**
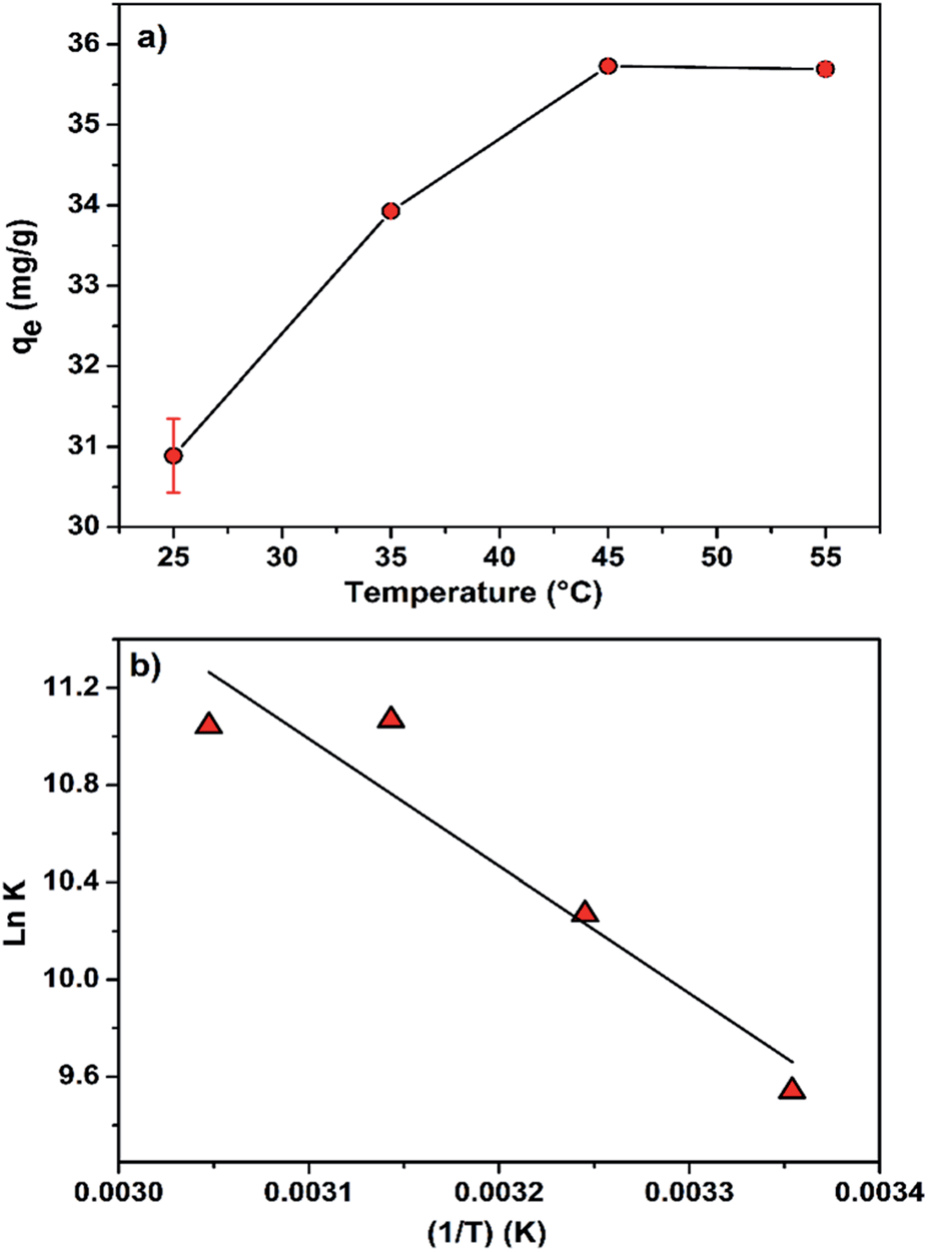
(a) Effect of the temperature on the adsorption capacity of MB by CMC-Alg/GO hydrogel beads and (b) Van't Hoff plot.

Regarding the effect of the temperature on the adsorption process (Fig. 8a), it is clearly observed that the adsorption capacity (mg g^−1^) increased with increasing temperature. This indicates that the adsorption process is controlled by an endothermic process.

The plot of ln *K versus* 1/*T* is presented in Fig. 8b and the values of various thermodynamic parameters are listed in Table 7. According to data in this table, the Δ*G*° exhibits negative values at a given temperature: −23.94 (298 K), −26.20 (308 K), −28.41 (318 K) and −30.72 (328 K), indicating that the adsorption process of MB onto CMC-Alg/GO beads required a low adsorption-energy, and that the MB uptake occurred favorably and spontaneously. From an energetic point of view, the favorability of an adsorption process is well established as the Δ*G*° becomes more negative.^24^ In another word, more Δ*G*° is negative more the adsorption process occurred favorably. The positive value of Δ*H*° (43.5 kJ mol^−1^) indicates that the adsorption process is endothermic in nature. Additionally, the positive value of Δ*S*° (0.226 kJ mol^−1^ K^−1^), proved a good affinity between the MB molecules and the CMC-Alg/GO adsorbent surface, and that a higher disorder tendency at the solid–solution interface occurred during the adsorption process.^16^ With the above background in mind, we suggest that both physisorption and chemisorption mechanisms are both involved in the adsorption of MB onto CMC-Alg/GO hydrogel beads with a predominance of the physisorption at some extent.

**Table:** Thermodynamic parameters for adsorption of MB on the CMC-Alg/GO beads

*T* (K)	*q*_e_ (mg g^−1^)	Δ*G* (kJ mol^−1^)	Δ*H* (kJ mol^−1^)	Δ*S* (kJ mol^−1^ K^−1^)	*R* ^2^	RMSE
298	30.888	−23.94	43.5	0.226	0.949	0.280
308	33.928	−26.20				
318	35.730	−28.41				
328	35.692	−30.72				

### 3.6. Desorption and reuse

**Fig. 9:**
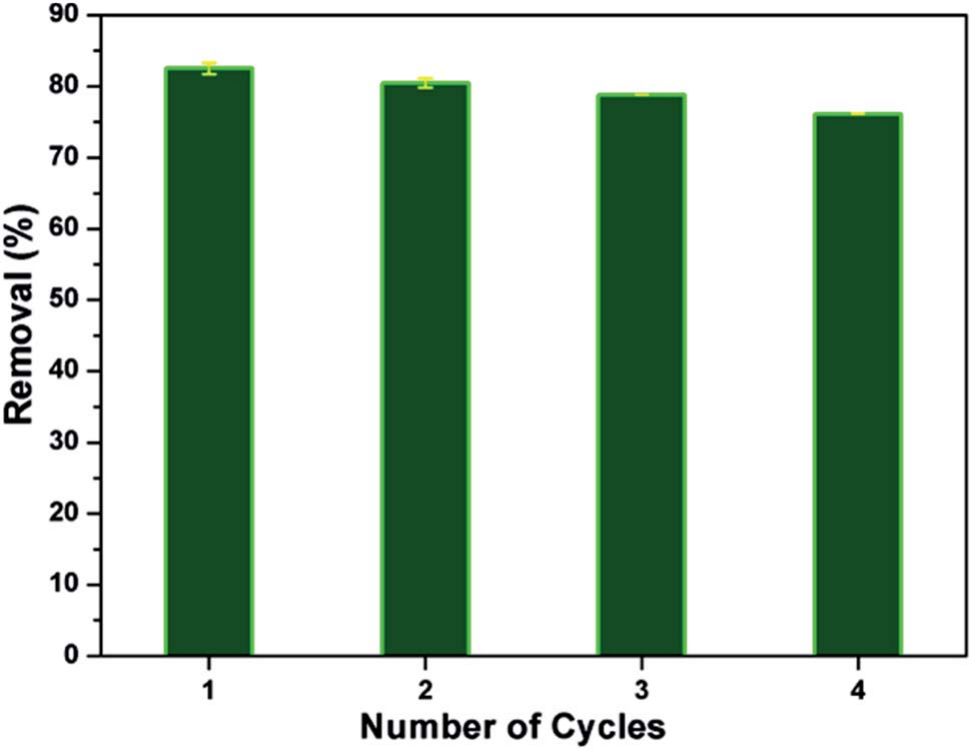
Removal efficiency of MB onto wet CMC-Alg/GO beads in fourth cycles.

In view of the industrial application, regeneration and reusability are key factors for the adsorption process. The adsorbent should exhibit a good reusability without significant loss in its affinity towards pollutants. In the present study, about 83% of MB dye has been desorbed as well as the reusability of the CMC-Alg/GO bead was investigated in fourth consecutive adsorption–desorption cycles, as shown in Fig. 9 Results obtained revealed that the as-prepared CMC-Alg/GO hydrogel beads is readily recyclable/reusable without significant drop in its uptake ability since the removal efficiency was maintained higher (76.07%) even after fourth successive batches adsorption–desorption cycles. Similar trend was also observed by T. Huang *et al.*^36^ when using magnetic graphene oxide modified zeolite for the effective removal of MB from aqueous solution. These authors reported a drop in the removal efficiency of MB from 89.59 to 76.86% after five consecutive cycles.

### 3.7. Adsorption mechanism

**Scheme 1:**
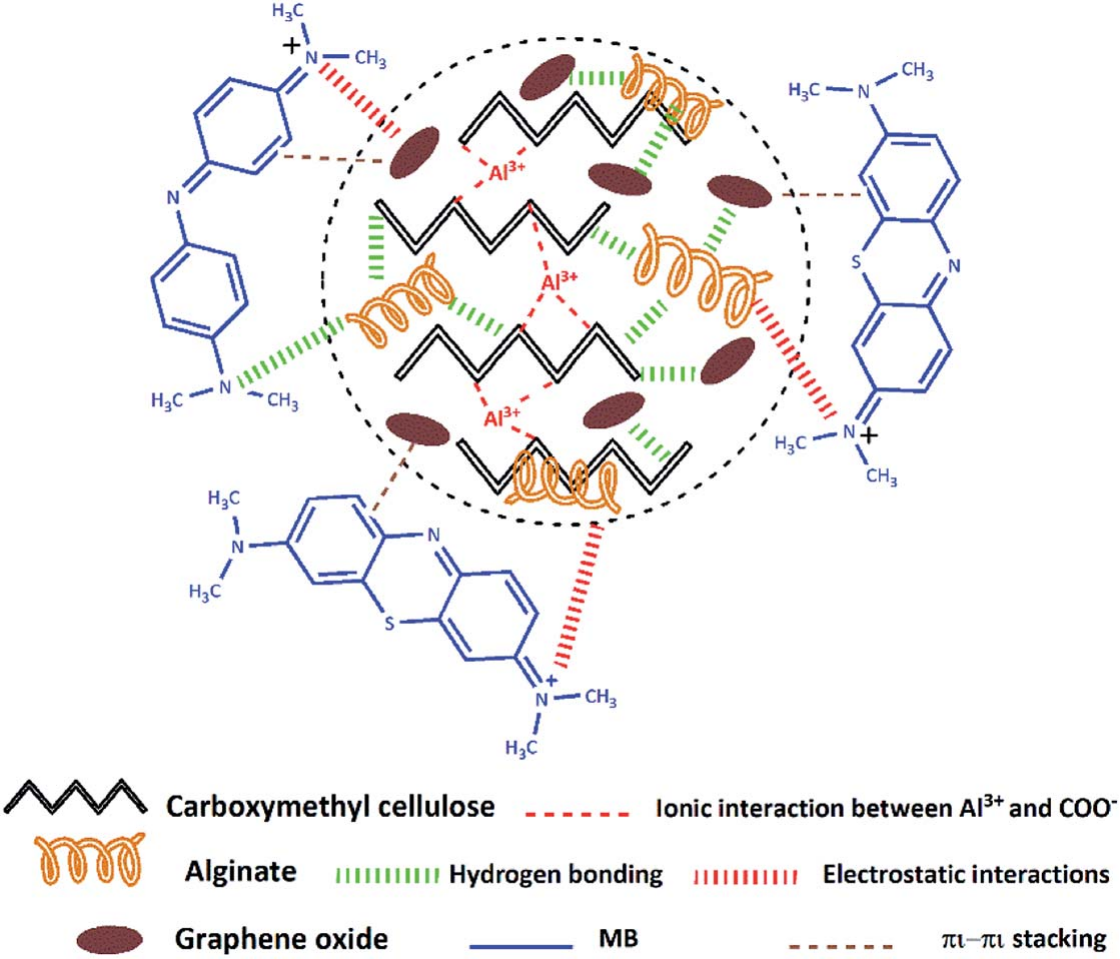
Suggested mechanism of MB adsorption onto CMC-Alg/GO hydrogel beads.

The adsorption mechanism of a contaminant on an adsorbent is depending on various factors, counting the adsorbent properties, the adsorbate nature, and the probable adsorbate/adsorbent interactions.^16^ Based on the above experimental studies as well as FTIR analysis (Fig. S2, ESI†) before and after MB adsorption on the CMC-Alg/GO hydrogel beads, a scheme was suggested to illustrate the MB adsorption mechanism (Scheme 1).

As we previously discussed and based on FTIR results (Fig. S2, ESI†) we can reasonably conclude that the functional groups present in CMC-Alg/GO hydrogel beads are involved in the adsorption of MB molecules *via* both hydrogen bonding and electrostatic interaction. Additionally, as MB molecules contain C=C double bonds and benzene rings with π electrons, it is favorably that MB molecule interact with GO through π–π stacking. With the above background in mind, we can reasonably conclude that both electrostatic interactions, π–π stacking and hydrogen bonding are presumably involved in the adsorption mechanism. Similar findings were also reported in previous works.^16^

### 3.8. Comparison with other adsorbents

According to the literature, several adsorbents have been investigated for MB removal from aqueous solution.^8,29^ The competitiveness of our adsorbent was examined against the other reported adsorbents as illustrated in Table 8. As shown in Table 8, the proposed adsorbent showed competitive adsorption ability and affinity towards MB with respect to the other adsorbents. Furthermore, the developed adsorbent is a novel, competitive, effective, practical and inexpensive.^37^ Hence, the prepared CMC/Alg-GO beads are highly recommended as efficient adsorbent due to their easy preparation, easy recovery and reusability which is favourable for treatment of effluents containing dyes, such as textile industry effluents.

**Table:** The removal (%) and the adsorption capacity of MB using various adsorbents

Adsorbents	*q*_m_ (mg g^−1^)	Removal (%)	Ref.
Alginate/almond peanut biocomposite	22.8	90	38
Magnetic graphene-carbon nanotubes composite	65.79	—	24
Polyamide-vermiculite nanocomposites	76.42	99	31
Guar gum-*g*-(acrylic acid-*co*-acrylamide-*co*-3-acrylamido propanoic acid)	32.1	—	28
CMC-Alg/GO hydrogel beads	78.5	96.2	Present work
CMC/kC/AMMT (1 : 1 : 0.4 ratio)	12.25	92	19
Chitosan/sepiolite composite	40.986	—	10
Salecan-*g*-PAI	107.1	—	37
H_2_SO_4_ crosslinked magnetic chitosan nanocomposite beads	20.408	—	3
CA-MWCNT-COOH	100.7	75.5	33
CAB biobeads	23	—	2

## 4. Conclusions

In this study, novel eco-friendly CMC-Alg/GO hydrogels beads were synthesized by an easily method and evaluated as a green adsorbent for the efficient removal of MB. The efficiency of the prepared hydrogel was significantly influenced by solution pH, initial MB concentration, contact time and temperature. The response surface methodology involving Box–Behnken design was effectively used to investigate the influence of three selected independent variables and to determine the optimal adsorption conditions. Based on RSM-BBD optimization, percentage removal of the MB molecule decreased with increase in initial dye concentration while it increased with increase in bioadsorbent dose and pH. The developed quadratic model represents adequately the response surface based on the adjusted determination coefficient (*R*_Adj_^2^ = 0.993). The optimized values, at which the maximum removal rate (96.22 ± 2.96) was reached, are got at pH = 9.5, initial MB concentration (15 mg L^−1^) and adsorbent dosage (0.6 g). The adsorption experimental data was well described by the Freundlich model (*R*^2^ = 0.975). Kinetics data suggested that the MB adsorption process on CMC-Alg/GO is predominant by the pseudo-second order adsorption mechanism (*R*^2^ = 0.999). Thermodynamic study showed that the adsorption process was spontaneous and endothermic in nature. Additionally, the desorption and reusability studies of the CMC-Alg/GO adsorbent under optimal condition showed that the adsorbent was easily recyclable up to four cycles with a slight decrease in its uptake ability. Consequently, the prepared hydrogels can be applied as stable, eco-friendly, efficient and reusable adsorbents for wastewater treatment.

## Conflicts of interest

The authors declare that there is no conflict of interest regarding the publication of this paper.

## Supplementary Material

RA-009-C9RA06450H-s001
