# Supplementary material for: Response surface methodology for optimization of methylene blue adsorption onto carboxymethyl cellulose-based hydrogel beads: adsorption kinetics, isotherm, thermodynamics and reusability studies
Source: RSC Adv. 2019 Nov 20;9(65):37858–69. doi: 10.1039/c9ra06450h (PMC9075779; doi:10.1039/c9ra06450h)
Supplement: RA-009-C9RA06450H-s001 [file RA-009-C9RA06450H-s001.pdf]

Electronic Supplementary Material (ESI) for RSC Advances.
This journal is © The Royal Society of Chemistry 2019

Electronic Supporting Information (ESI)

Response surface methodology for optimization of methylene blue adsorption onto carboxymethyl cellulose-based hydrogel beads: Adsorption kinetic, isotherm, thermodynamic and reusability studies

Dalia Allouss,[a] Younes Essamlali,[b] Othmane Amadine[b], Achraf Chakir[a] and Mohamed Zahouily[*a,b]

Preparation of CMC-Alg/GO hydrogel beads

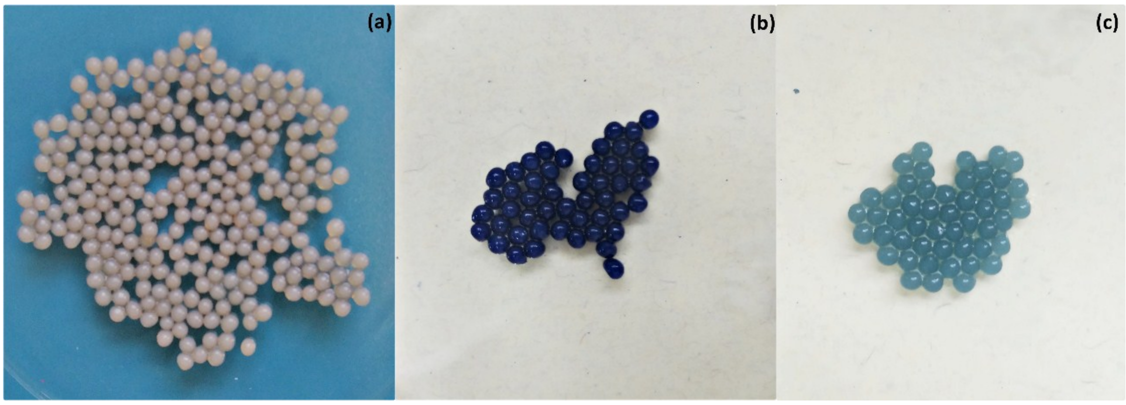

Fig.S1 Digital Images of wet CMC-Alg/GO beads (a) before MB adsorption, (b) after MB adsorption and (c) after MB desorption.

Characterization of CMC-Alg/GO beads

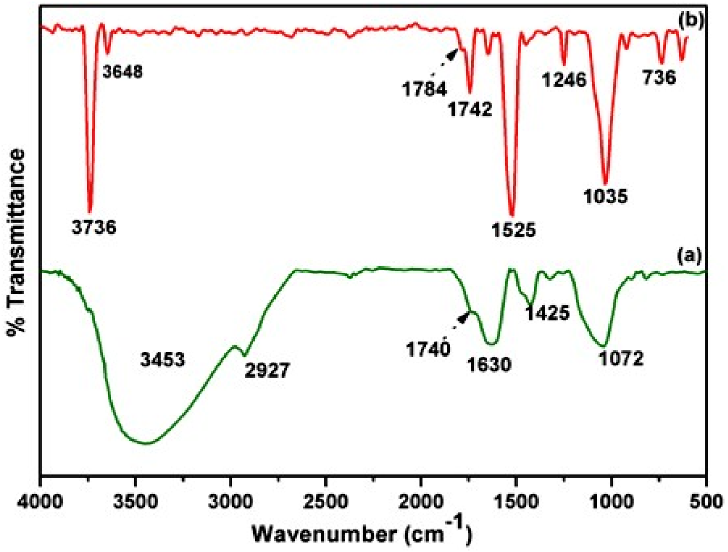

Fig.S2 ATR-FTIR spectra of CMC-Alg/GO (a) before and (b) after MB removal.

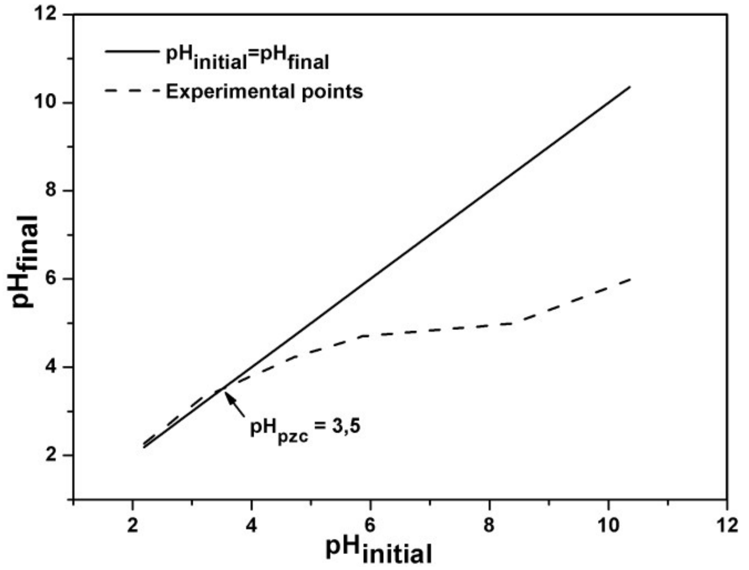

Fig.S3 pH_{pzc} of wet CMC-Alg/GO beads.

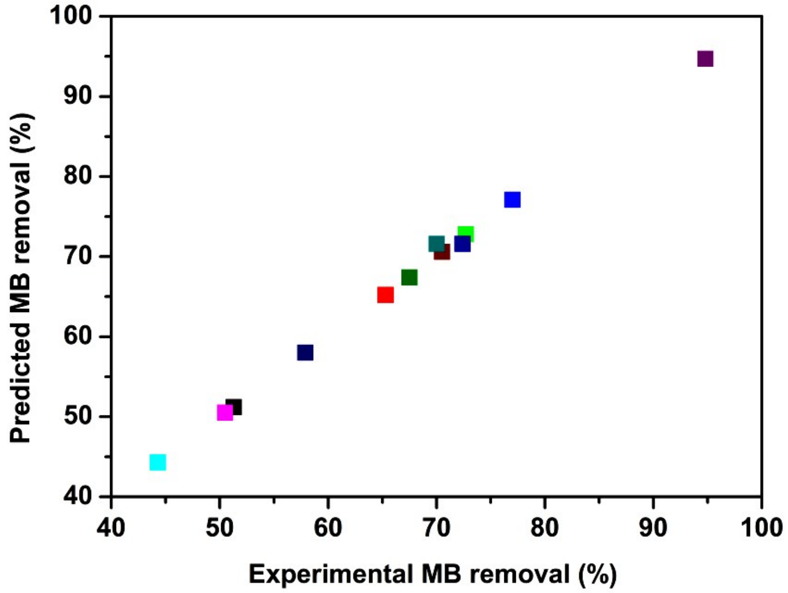

Fig.S4 Plot of the experimental and model-predicted values of the MB removal rate (%).

Optimization of process parameters using response surface methodology

The ANOVA analysis expresses the equation and relationship between the response and significant variables. Thus, p-values inferior than 5% announce that the model has statistically significance and values superior than 10 % indicate the variables of the model are statistically not significant. In order to verify the validity of both regression models (% Removal and adsorption capacity mg/g), two experiments (run 7 and 8) were deactivated because of their high distance between the experimental and predicted values by the models. The test of student was statistically significant (p-value (%) <5%). The analysis of variance (ANOVA) (**Table S2**) revealed for both responses that the models were valid which was explained by the lower p-value (%) than 5%. There is an excellent correlation between the experimental and predicted values as proved by nearness between R^2 and adjusted R^2 value.

Table S1 Analysis of variance (ANOVA) for quadratic model for Methylene Blue adsorption

Response	Source	Sum of squares	D_f	Mean square	F-value	P-value (%)	Comment
MB removal (%)	Regression	2099.47	9	233.275	187.53	0.0622	SD = 1.14 %
	Residuals	3.92	3	1.31	-	-	CV = 1.6%
	Lack of fit	0.08	1	0.08	0.0417	85.7	R^2 = 0.998
	Pure error	3.84	2	1.92	-	-	$R^2_{adjusted}$ = 0.993
MB uptake (mg/g)	Regression	3629.04	9	403.27	39.34	0.587	SD = 3.20 mg/g
	Residuals	30.75	3	10.25	-	-	CV = 8.14%
	Lack of fit	22.11	1	22.11	5.19	15.2	R^2= 0.992
	Pure error	8.64	2	4.32	-	-	$R^2_{adjusted}$ = 0.966

Table S2. Estimated regression coefficients for MB removal efficiency (%) in coded units

Terms	Coefficients	SE coefficient	t-value	P-value (%)
Constant	71.600	0.659	108.490	0.01
X_1	4.575	0.571	8.000	0.407
X_2	8.375	0.404	20.720	0.0246
X_3	-3.675	0.571	-6.430	0.763
X^2_1	-16.987	0.719	-23.620	0.0166
X^2_2	11.962	0.719	16.630	0.0473
X^2_3	-10.887	0.719	-15.140	0.0626
X_1X_2	-2.425	0.571	-4.240	2.40
X_1X_3	1.475	0.989	1.490	23.3
X_2X_3	-9.975	0.571	-17.450	0.0410

Table S3. Estimated regression coefficients for MB removal capacity (mg/g) in coded units

Terms	Coefficients	SE coefficient	t-value	P-value (%)
Constant	39.30	1.84	21.26	0.0228
X_1	-13.700	1.600	-8.56	0.335
X_2	4.088	1.131	3.61	3.65
X_3	16.525	1.600	10.32	1.194
X^2_1	-5.200	2.014	-2.58	8.2
X^2_2	6.300	2.014	3.13	5.2
X^2_3	-9.025	2.014	-4.48	2.07
X_1X_2	-3.550	1.600	-2.22	11.3
X_1X_3	-11.650	2.772	-4.20	2.46
X_2X_3	-2.425	1.600	-1.51	22.7

MB adsorption capacity response:

As exposed in **Table S4**, the linear model terms (adsorbent dose (X_1), pH (X_2) and initial dye concentration (X_3)), quadratic model terms (X_1^2, X_2^2 and X_3^2) and interactive model term (X_1X_3) are statistically significant. According to the BBD results, the final model is given by equation below:

$$\hat{Y}_2 = 39.30 - 13.7\,X_1 + 4.088\,X_2 + 16.525\,X_3 - 5.2\,X_1^2 + 6.3\,X_2^2 - 9.025\,X_3^2 - 11.65\,X_1X_3$$

It is obvious that the capacity of MB adsorbed gets increased with increase in the initial MB concentration which enhances the interaction between MB and CMC-Alg/GO beads (**Fig. S5.a and S5.b**).

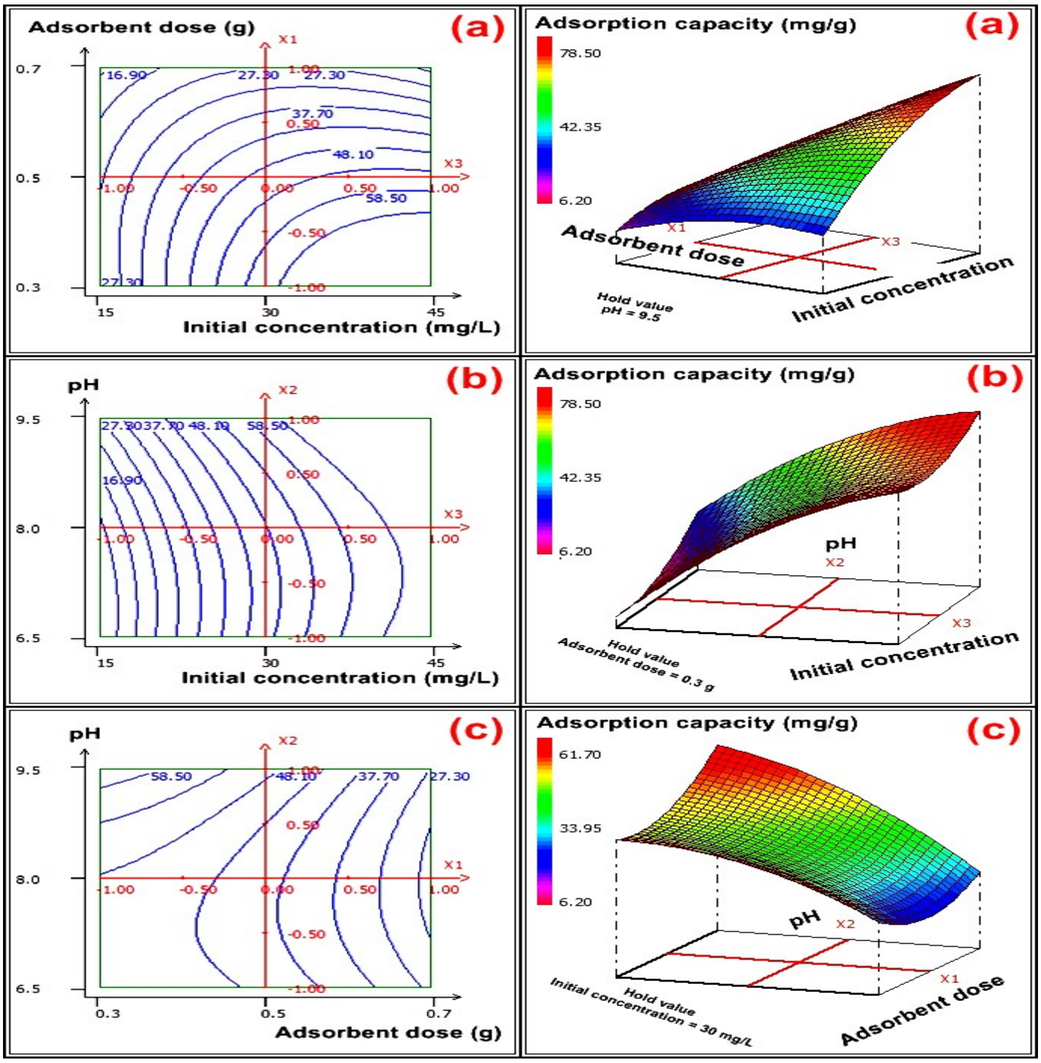

Fig. S5 2D-3D response surface plots of the effect (a) the adsorbent dose and initial concentration, (b) the pH and initial concentration and (c) the pH and adsorbent dose on MB adsorption capacity (mg/g) by CMC-Alg/GO beads.
